# A data set on anthropometric measurements and degree of discomfort of physically disabled workers for ergonomic requirements in work space design

**DOI:** 10.1016/j.dib.2020.105420

**Published:** 2020-03-12

**Authors:** Mahmoud Z. Mistarihi

**Affiliations:** Department of Industrial Engineering, Yarmouk University, Irbid, Jordan

**Keywords:** Anthropometric measurements, Discomfort questionnaire, Physically disabled workers, Work space design, Ergofellow software

## Abstract

Workers movements and their body mechanics during work, design of tools as well as work layout is important to fit the task to match differences between human capabilities. The data set examined 40 physically disabled workers from different areas and background in Jordan. It consists of anthropometric measurements that are categorized into 7 key measures namely: weight, stature, hip height, knee height, elbow height, hand length and Elbow-fingertip length. Also, it includes information on the most parts that cause pain for the same participants using the discomfort questionnaire. The dataset supports the article " An integration of a QFD model with Fuzzy-ANP approach for determining the importance weights for engineering characteristics of the proposed wheelchair design"[1]. The obtained dataset can also be used to support an ergonomic and bio-mechanical evaluation performance of physically disabled workers as well as using it in conjunction with ISO standards for equipment design and safety. Moreover, This dataset is useful for optimizing the dimensions’ design for the physically disabled workplace as well as preparing the House of Quality to prioritize the final design requirements.

Specifications table

**Table:** 

Subject area	*Ergonomics and human engineering*
More specific subject area	*Anthropometry and work space design*
Type of data	*Tables, figures*
How data was acquired	*Measurement instruments, Ergofellow software, Discomfort questionnaire*
Data format	*Raw and analyzed*
Parameters for data collection	*40 physically disabled workers (20–40 years age range)were considered for the analysis and the anthropometric measurements are categorized into 7 key measures namely: weight, stature, hip height, knee height, elbow height, hand length and Elbow-fingertip length*
Description of data collection	*The anthropometric data were obtained from 40 physically disabled workers using: Stadiometers, Bicondylar Harpenden skinfold caliper and Digtal Bathroom Scale. Also, systematic measurements of the human body using Anthropometry component within the Ergofellow software as well as the discomfort questionnaire.*
Data source location	*Jordan.* *Latitude and longitude: 30.5852° N, 36.2384° E*
Data accessibility	*Data are included in this article*
Related research article	Mistarihi M et al., 2020 "An integration of a QFD model with Fuzzy-ANP approach for determining the importance weights for engineering characteristics of the proposed wheelchair design, Applied Soft Computing, Volume 90, https://doi.org/10.1016/j.asoc.2020.106136.

## Value of the data

• May be used in Ergofellow software to identify musculoskeletal disorders that may effect on most common work postures for physically disabled workers.
• It can be used in Quality Function Deployment (QFD) applications to transfer the Voice Of Customers (VOC) into Engineering Characteristics for a product such as a disabled wheelchair.
• This data is of value to those who are doing research on product development especially during designing product ergonomically.
• The data set can be used by researchers to calculate stress, strain and displacement analysis using SOLIDWORK for any related product design such as a disabled wheelchair.

## 1 Data

**Fig. 1:**
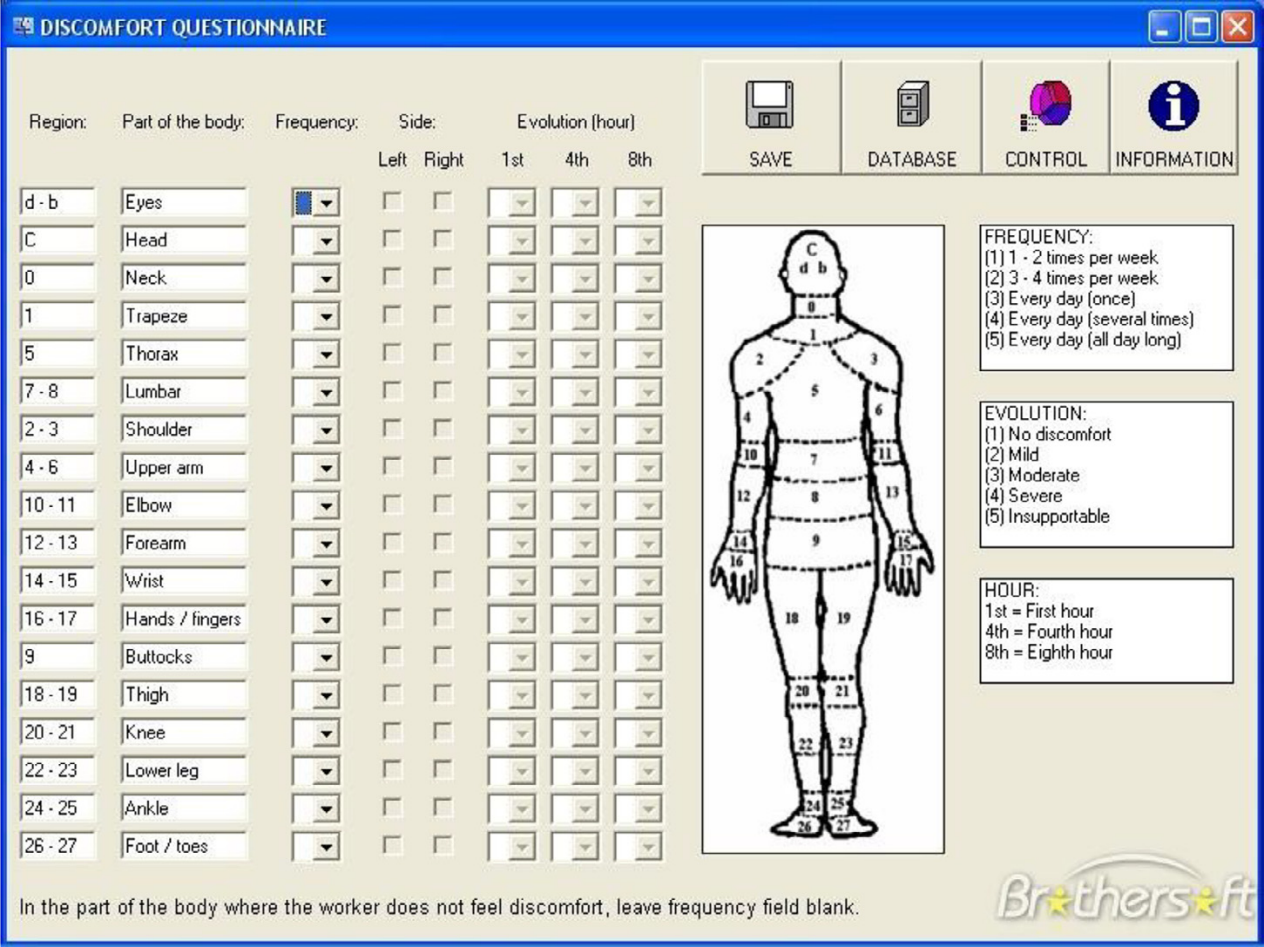
The discomfort questionnaire in ergo fellow software.

**Fig. 2:**
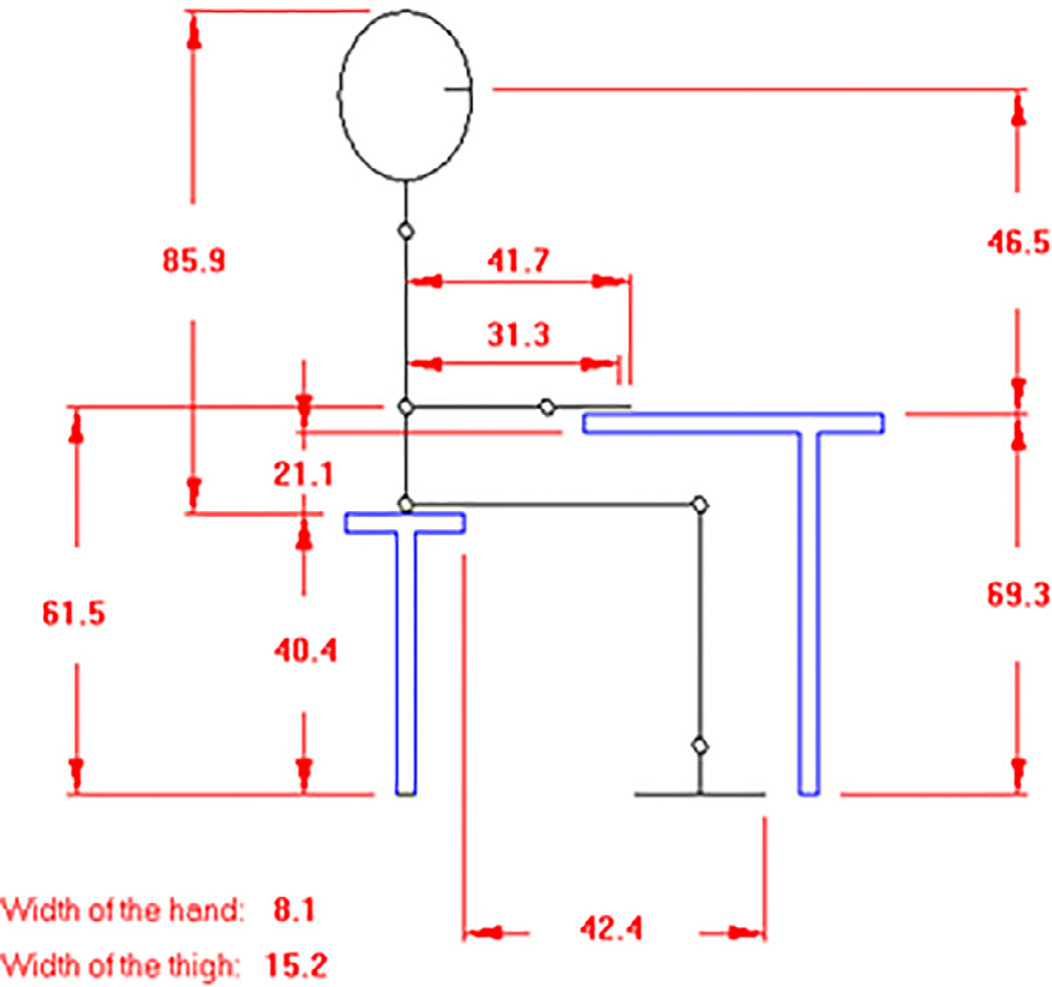
The statistical summaries of body measurements (cm) for physically disabled workers working in sitting position.

**Fig. 3:**
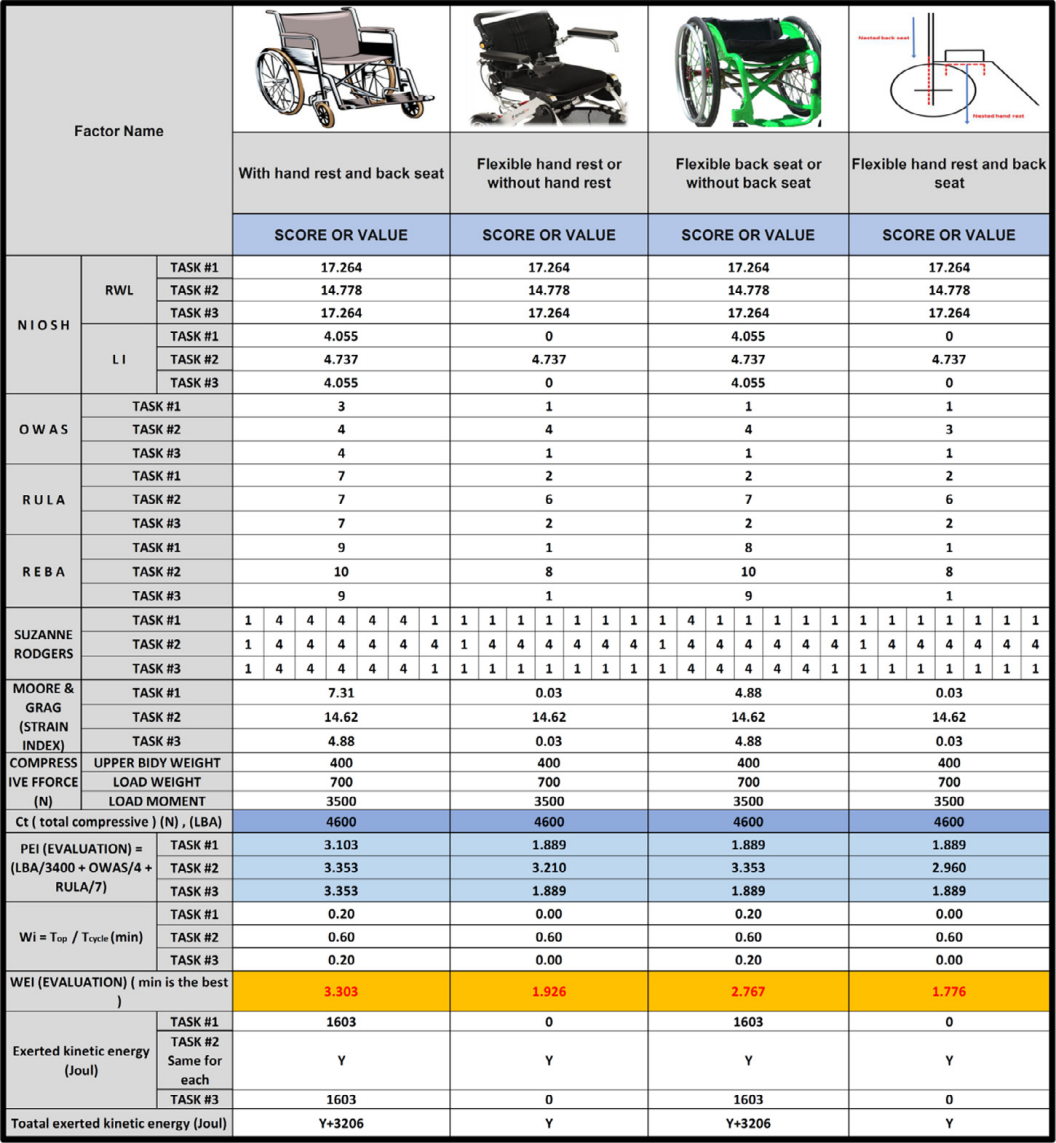
Comparison between different disabled wheelchairs design according to Posture Evaluating Index (PEI), as well as Work Evaluation Index (WEI).

The data shared here are tables and figures presenting information on anthropometric measurements and the discomfort questionnaire of the human body for physically disabled workers. Also, the data supports a research paper in product design, development and assessment [1]. Anthropometry is the science which concerns with the human body dimensions and physical characteristics. Human factors engineers are always in need to Anthropometry to improve their everyday consumer products to enhance the work environment, making it safer and more comfortable [2]. The data are intended for use in conjunction with ISO standards for equipment design and safety. Characteristics of the measuring devices used in getting the anthropometric data are shown in Table 1. Table 2 illustrates the degree of complain at different parts of the body related to the discomfort questionnaire for the 40 physically disabled workers. Table 3 shows anthropometric measurements with 7 key measures namely: weight, stature, hip height, knee height, elbow height, hand length and Elbow-fingertip length. Descriptive statistics for anthropometric measurements of physically disabled workers are presented in Table 4. Fig. 1 presents the discomfort questionnaire in ergofellow that can identify parts of muscle or joint which are more effected based on the Nordic Body Map [3]. Figs. 2 shows the statistical summaries of body measurements for physically disabled workers working in sitting position. Fig. 3 demonstrates a comparison between different disabled wheelchairs designs according to Posture Evaluating Index (PEI), as well as Work Evaluation Index (WEI).

**Table 1:** Characteristics of the measuring devices used in getting the anthropometric data.

Tool	Characteristics
Stadiometers	seca Portable model 213
Bicondylar Caliper	*Holtain Bicondylar Caliper measuring range 0mm-140* *mm*, model 604
skinfold caliper	*Harpenden skinfold caliper with a precision of +/- 0.2* *mm*, model C-120B
Scale	*EatSmart Precision CalPal Digtal Bathroom Scale, model ESBS-52*

**Table 2:** The degree of complaint at different parts of the body related to the discomfort questionnaire.

Number	location	Average degree of compliant
		1–5
0	Pain in the upper neck	4
1	Pain in the lower neck	4
2	Pain in the left shoulder	3
3	Pain in the right shoulder	3
4	Pain in the left upper arm	2
5	Pain in the back	5
6	Pain in the right upper arm	3
7	Pain in the wrist	3
8	Pain in the buttock	2
9	Pain in the bottom	2
10	Pain in the left elbow	4
11	Pain in the right elbow	4
12	Pain in the left lower arm	3
13	Pain in the right lower arm	3
14	Pain in the left wrist	4
15	Pain in the right wrist	4
16	Pain in the left hand	3
17	Pain in the right hand	3
18	Pain in the left thigh	2
19	Pain in the right thigh	2
20	Pain in the left knee	3
21	Pain in the right knee	3
22	Pain in the left calf	4
23	Pain in the right calf	4
24	Pain in the left ankle	2
25	Pain in the right ankle	2
26	Pain in the left foot	4
27	Pain in the right foot	4

**Table 3:** Anthropometric measurements of physically disabled workers.

no	Weight (Kg)	Stature (cm)	Elbow-fingertip length (cm)	Elbow height (mm)	Hand length (mm)	Knee height (cm)	Hip height (cm)
1	65	170	44	114	166	53	100
2	54	175	45	120	178	52	106
3	73	166	42	110	173	50	92
4	85	163	41	109	169	46	90
5	77	170	43	113	170	50	100
6	67	167	45	109	168	49	98
7	53	162	44	100	167	47	96
8	85	166	41	107	170	50	93
9	80	165	43	107	170	50	90
10	47	155	42	104	167	45	88
11	50	167	43	112	173	50	93
12	73	169	44	110	164	50	96
13	69	165	42	109	162	55	98
14	72	158	41	107	165	49	93
15	50	170	44	114	170	53	100
16	75	163	43	104	167	50	98
17	75	162	41	104	166	50	92
18	58	170	43	109	173	54	102
19	66	162	42	103	165	50	90
20	76	153	40	102	164	45	87
21	65	170	42	114	166	50	99
22	54	163	45	120	178	52	106
23	73	166	44	109	173	46	93
24	85	175	41	110	169	53	90
25	79	170	44	112	168	52	99
26	67	166	45	109	168	50	98
27	53	162	44	97	167	47	96
28	83	167	40	107	170	50	93
29	77	165	43	107	172	49	91
30	53	160	42	100	167	47	88
31	49	167	43	112	173	50	93
32	70	169	43	113	162	52	95
33	70	160	43	106	160	53	99
34	72	155	41	107	165	49	93
35	50	170	41	112	169	53	100
36	75	163	43	104	168	50	98
37	75	162	41	104	166	50	91
38	58	169	43	111	171	52	99
39	66	163	44	104	165	52	93
40	76	157	40	101	166	45	88

**Table 4:** Descriptive statistics for anthropometric measurements of physically disabled workers.

No	Weight (Kg)	Stature (cm)	Elbow-fingertip length (cm)	Elbow height (mm)	Hand length (mm)	Knee height (cm)	Hip height (cm)
Mean	67.5	164.9	42.6	108.2	168.3	50	95.1
SD	11.2	5.0	1.4	5.0	3.9	2.5	4.7
5th% tile	49.9	155	40	100	162	45	88
95th% tile	85	170.3	45	114.3	173.3	53.1	102.2

## 2 Experimental design, materials, and methods

Physically disabled workers resident in different areas of Irbid governorate, Jordan were invited to take part in this data paper. From the 157 physically disabled workers who agreed to participate, 40 workers were selected using a simple random sampling. All of these participants were aware of the objective of doing such a research in reducing the musculoskeletal disorders via product design and development. Data was available for 40 actual participants in age range of 20–40 years old.

Anthropometric methods were used to measure the dimensions of physically disabled worker's body and some of its parts, as well as the correlations between these dimensions. The materials used to get the anthropometric data are: Stadiometers, Bicondylar Caliper, skinfold caliper and Scale. The characteristics of these measuring tools are shown in Table 1. Also, systematic measurements of the human body using Anthropometry component within the Ergofellow software [4] as well as the discomfort questionnaire.

The Cornell Musculoskeletal Discomfort Questionnaire (CMDQ) (Fig. 1) has been adapted here with Nordic Body Map. It is similar in many respects to the discomfort surveys used by the National Institute for Occupational Safety and Health (NIOSH) [5]. Actually, it divides body parts into numbering from 0 to 27 covering the whole body from neck to feet. It is based on previous postural discomfort surveys and it has high face validity. It is for research screening purposes and should not be used as a diagnostic instrument. The validity of the diagnosis in this data paper can be tested in any comparative examination of responses to clinical reports [6].

## Appendix Supplementary materials

Image, application 1

Image, application 2
