# Supplementary material for: A data set on anthropometric measurements and degree of discomfort of physically disabled workers for ergonomic requirements in work space design
Source: Data Brief. 2020 Mar 12;30:105420. doi: 10.1016/j.dib.2020.105420 (PMC7113429; doi:10.1016/j.dib.2020.105420)
Supplement: Supplementary file 2 [file mmc2.pdf]

The diagram below shows the approximate position of the body parts referred to in the questionnaire. Please answer by marking the appropriate box.

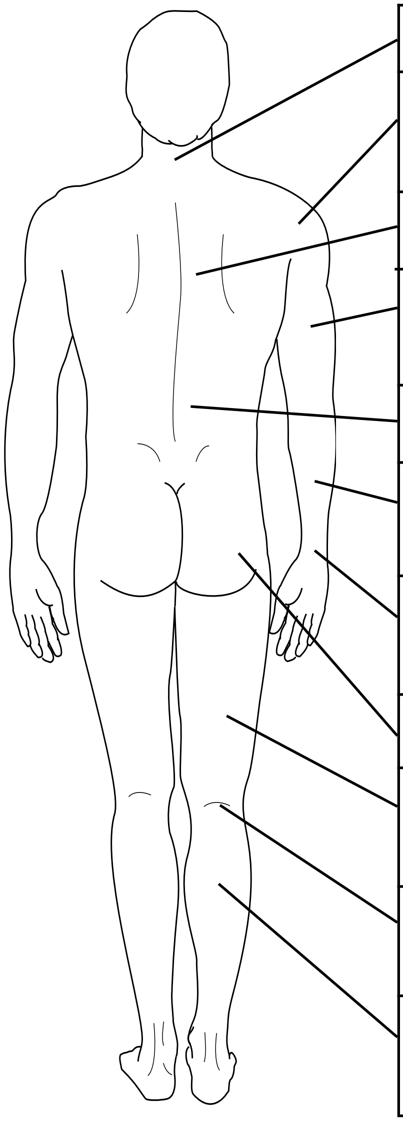

		During the last work <u>week</u> how often did you experience ache, pain, discomfort in:					If you experienced ache, pain, discomfort, how uncomfortable was this?			If you experienced ache, pain, discomfort, did this interfere with your ability to work?		
		Never	1-2 times last week	3-4 times last week	Once every day	Several times every day	Slightly uncomfortable	Moderately uncomfortable	Very uncomfortable	Not at all	Slightly interfered	Substantially interfered
Neck		☐	☐	☐	☐	☐	☐	☐	☐	☐	☐	☐
Shoulder	(Right)	☐	☐	☐	☐	☐	☐	☐	☐	☐	☐	☐
	(Left)	☐	☐	☐	☐	☐	☐	☐	☐	☐	☐	☐
Upper Back		☐	☐	☐	☐	☐	☐	☐	☐	☐	☐	☐
Upper Arm	(Right)	☐	☐	☐	☐	☐	☐	☐	☐	☐	☐	☐
	(Left)	☐	☐	☐	☐	☐	☐	☐	☐	☐	☐	☐
Lower Back		☐	☐	☐	☐	☐	☐	☐	☐	☐	☐	☐
Forearm	(Right)	☐	☐	☐	☐	☐	☐	☐	☐	☐	☐	☐
	(Left)	☐	☐	☐	☐	☐	☐	☐	☐	☐	☐	☐
Wrist	(Right)	☐	☐	☐	☐	☐	☐	☐	☐	☐	☐	☐
	(Left)	☐	☐	☐	☐	☐	☐	☐	☐	☐	☐	☐
Hip/Buttocks		☐	☐	☐	☐	☐	☐	☐	☐	☐	☐	☐
Thigh	(Right)	☐	☐	☐	☐	☐	☐	☐	☐	☐	☐	☐
	(Left)	☐	☐	☐	☐	☐	☐	☐	☐	☐	☐	☐
Knee	(Right)	☐	☐	☐	☐	☐	☐	☐	☐	☐	☐	☐
	(Left)	☐	☐	☐	☐	☐	☐	☐	☐	☐	☐	☐
Lower Leg	(Right)	☐	☐	☐	☐	☐	☐	☐	☐	☐	☐	☐
	(Left)	☐	☐	☐	☐	☐	☐	☐	☐	☐	☐	☐

© Cornell University, 1994
